# MAPK Activated Protein Kinase 3 Is a Prognostic-Related Biomarker and Associated With Immune Infiltrates in Glioma

**DOI:** 10.3389/fonc.2021.793025

**Published:** 2021-12-06

**Authors:** Jing Ren, Jinmin Sun, Mengwei Li, Zifan Zhang, Dejun Yang, Haowei Cao

**Affiliations:** Jiangsu Key Laboratory of Brain Disease and Bioinformation, Research Center for Biochemistry and Molecular Biology, Xuzhou Medical University, Xuzhou, China

**Keywords:** MK3, prognosis, biomarker, immune infiltrate, glioma

## Abstract

Glioma is the most common primary brain tumor that causes significant morbidity and mortality. *MAPK activated protein kinase 3* (*MAPKAPK3*/*MK3*) is a serine/threonine protein kinase regulating various cellular responses and gene expression. However, the role of *MK3* in tumor progress, prognosis, and immunity for glioma remains unclear. Here, we determined the expression and prognostic values of *MK3.* We further analyzed the correlation of *MK3* expression with immune infiltrations by using the biochemical methods and bioinformatic approaches with available databases. We find that *MK3* is aberrantly upregulated in glioma. In addition, the higher *MK3* expression is closely linked to the poor clinicopathologic features of glioma patients. Importantly, *MK3* expression is negatively correlated with the prognosis of patients with glioma. Mechanistically, we demonstrated that the correlated genes of *MK3* were mainly enriched in pathways that regulate tumor immune responses. The *MK3* level was significantly associated with tumor-infiltrating immune cells and positively correlated with the majority of tumor immunoinhibitors, chemokines, and chemokine receptors in glioma. Thus, these findings suggest the novel prognostic roles of *MK3* and define *MK3* as a promising target for glioma immunotherapy.

## Introduction

Glioma is known as the most common primary brain and spinal cord tumor (1), with an estimated annual incidence rate of 6.57 per 100,000 persons in the United States (2). Despite their relatively rare rate, glioma usually causes significant morbidity and mortality because of the low treatment success rate and poor overall survival (OS) rate. Among the newly diagnosed glioma, glioblastoma (GBM) is the most malignant form of brain cancer accounting for about 50% (3), with a 5-year survival rate of approximately 5% (4). So far, the main therapeutic options for glioma patients include surgery resection (5), chemotherapy (6), radiotherapy (7), and immunotherapy (8). However, the glioma, especially GBM, still remains incurable with poor prognosis, which imposes enormous pressure on society, although some clinically relevant epigenetic biomarkers such as IDH status, *O*-6-methylguanine-DNA methyltransferase (MGMT) promoter methylation status, histone code, and chromatin organization have been used for classification of glioma and treatment decisions (9). Thus, searching for the novel specific biomarker for advancing the prognosis of glioma remains an imperative challenge.

*MAPK activated protein kinase 3* (*MAPKAPK3*/*MK3*) was first reported in 1996, located on human chromosome band 3p21.2 (10). *MK3* belongs to the Ser/Thr protein kinase family, which functions as *mitogen-activated protein kinase* (*MAPK*)-activated protein. *MK3* shares a highly similar sequence to *MAPK activated protein kinase 2* (*MAPKAPK2*/*MK2*), possessing 72% nucleotide and 75% amino acid identity (11). *MK3* is primarily activated by the *MAPKs p38 α/β* (12), *ERK1/2*, and *JNK1/2* (10). *MK3* plays an important role in regulating cytokine production (13), endocytosis (14), interferon therapy (15), autophagy (16), inflammation (17), intimal hyperplasia (18), chromatin remodeling (19), and transcription regulation (20). *MK3* has been associated with several diseases, such as glomerulonephritis (21), skin disease (22), influenza A virus infection (23), and diabetes (24).

A previous study has reported that *MK3* could act as a reliable prognostic indicator for colorectal cancer patients (25) as well as regulate NK cell cytotoxicity and CD4 T-cell development (23). However, the expression, clinical significance, biological roles, and potential molecular mechanisms of *MK3* in glioma have yet not been investigated. Here, we revealed that *MK3* was aberrantly overexpressed in glioma tissues and cell lines. We reported that the *MK3* expression was closely associated with the poor clinicopathologic features and prognosis of glioma. Through the function and pathway enrichment analyses, we demonstrated that the correlated genes of *MK3* were mainly enriched in immune regulatory pathways. Finally, we recovered the close correlation of *MK3* expression with immune infiltration, immune-related genes, and immune checkpoints in glioma. Together, our study deciphered the essential role of *MK3* in glioma prognosis and tumor immunoregulation.

## Materials and Methods

### Human Tissues

We collected 92 glioma tissues that were identified by the pathologists according to the 2016 WHO classification criteria from the Department of Pathology of the Affiliated Hospital of Xuzhou Medical University between 2016 and 2017. We obtained the ethical review and approval from the institutional ethics committee of Affiliated Hospital of Xuzhou Medical University (ethical review no. XYFY2018-KL056-01).

### Cell Culture

Human GBM cell lines (U118, U87, U251, T98G, and LN229), human normal brain glial cells (HEB), and normal human astrocyte (NHA) were originally obtained from the American Type Culture Collection (ATCC). All these cell lines were cultured with Dulbecco’s modified Eagle’s medium (DMEM; KeyGen Biotech, China) containing 10% fetal bovine serum (FBS, Takara, Japan) at 37°C within a humid atmosphere containing 5% CO_2_.

### Gene Expression Analysis

We analyzed the *MK3* expression with the “Single Gene Analysis” module of Gene Expression Profiling Interactive Analysis (GEPIA) web (http://gepia.cancer-pku.cn/) and observed the differential expression of *MK3* between tumor and adjacent normal tissues for the different tumors or specific tumor subtypes of The Cancer Genome Atlas (TCGA). RNA-sequencing (RNA-seq) data of 689 GBM and low-grade glioma (LGG) tissues from TCGA dataset (https://www.cancer.gov/about-nci/organization/ccg/research/structural-genomics/tcga) and 1,157 normal tissues from Genotype-Tissue Expression (GTEx) (https://www.genome.gov/Funded-Programs-Projects/Genotype-Tissue-Expression-Project) were used for *MK3* expression analyses. RNA-seq data of 413 glioma tissues (batch I) and 273 glioma tissues (batch II) datasets after deletion of incomplete data from the Chinese Glioma Genome Atlas (CGGA) dataset were also utilized for the expression analyses. In addition, we analyzed the expression of *MK3* in glioma by using the Gene Expression Omnibus (GEO) database, GSE4290, and GSE7696 (https://www.ncbi.nlm.nih.gov/geo/). t-Test was used for the expression analyses by using R package “ggplot2.”

### Western Blotting

Cells were lysed with radioimmunoprecipitation assay (RIPA) extraction reagent [50 mM of Tris-HCl, pH 8.0, 150 mM of NaCl, 0.5% sodium deoxycholate, 1% NP-40, and 0.1% sodium dodecyl sulfate (SDS)] containing the protease inhibitors (Roche, Germany). Total protein was separated using 10% polyacrylamide gel electrophoresis and transferred to NC membrane (Millipore, USA). The membrane was blocked with 5% bovine serum albumin (BSA) in TBST for 1 h at room temperature and then incubated with the primary antibodies against *MK3* (1:200, Santa Cruz, USA) and GAPDH (1:10,000, ProteinTech, USA) at 4°C overnight. Then the membrane was washed three times using 0.1% TBST buffer and incubated with the horseradish peroxidase (HRP)-conjugated secondary antibodies for 1 h at room temperature, followed by three times TBST buffer washing. Finally, the signal was detected with enhanced chemiluminescence reagent.

### Immunohistochemistry

The 4-μm-thick glioma sections were stained with a specific primary antibody against *MK3* (1:50, Santa Cruz, USA) at 4°C overnight. Then the sections were incubated with secondary antibody (ZSGB-BIO, China) for 1 h and DAB for 2–5 min at room temperature, followed by hematoxylin staining. The images were acquired by Olympus microscopy and scored by two experienced pathologists without knowing the patients’ characteristics. Scores were calculated based on the intensity and percentage of positive tumor cells within the whole tissue, which were evaluated by using the German semiquantitative scoring method. The intensity score of cytoplasmic staining of each specimen was defined as follows: 0, negative; 1, weak; 2, moderate; and 3, strong. The percentage of positive cells was evaluated with 0.0% staining; 1, 1%–24 % staining; 2, 25%–49% staining; 3, 50%–74% staining; and 4, 75%–100% staining. The final immunoreactive score was calculated by the multiplication of the intensity scores and proportion scores.

### Survival Analysis

We obtained the survival and clinical phenotype data of each sample from TCGA and CGGA datasets. The Kaplan–Meier (KM) curve analyses were conducted by the R package “survival” and “survminer.” Cox analysis was used for survival analyses by using the R package “survival.” The receiver operating characteristic (ROC) analysis was performed with the R package “pROC.”

### Univariate and Multivariate Cox Analyses

Univariate and multivariate Cox analyses were conducted by using the R package “survival.” The WHO grade, IDH status, 1p/19q codeletion, primary therapy outcome, gender, age, and *MK3* expression were included in these analyses.

### Protein–Protein Interaction Network of *MK3* Analysis

We searched the *MK3* with the query of protein name (“*MK3*”) and organism (“Homo sapiens”) on the STRING website (https://string-db.org/). The basic setting parameters were the network type (“full STRING network”), meaning of network edges (“evidence”), active interaction sources (“experiments”), minimum required interaction score (“low confidence (0.150)”), and max number of interactors to show (“no more than 50 interactors in 1^st^ shell”).

### Gene Ontology and Kyoto Encyclopedia of Genes and Genome Analyses

Gene expression data of GBM and LGG in HTSeq-Counts were downloaded from TCGA website for further analysis. The correlated genes of *MK3* were screened with Pearson’s correlation coefficients (|*r*| >0.4 and *p* < 0.001) with R package “deseq2.” Gene Ontology (GO) and Kyoto Encyclopedia of Genes and Genome (KEGG) analysis were conducted on *MK3* correlated genes with R package “clusterProfiler” to identify the possible biological functions and signaling pathways affected by *MK3*. The biological process (BP), cellular component (CC), and molecular function (MF) were applied in GO analysis.

### Gene Set Enrichment Analysis

The Gene Set Enrichment Analysis (GSEA) was performed to dissect the cancer-related pathways by using the MSigDB Collection (c2.cp.v7.2.symbols.gmt) of the clusterProfiler R package. The gene sets with normal *p*. adjust value and false discovery rate (FDR) *q* value both less than 0.05 were considered as significantly enriched.

### Single-Cell RNA-Sequencing Analysis

We obtained the single-cell RNA-seq data from the Single Cell^BETA^ PORTAL website (https://singlecell.broadinstitute.org/single_cell/study/SCP393/single-cell-rna-seq-of-adult-and-pediatric-glioblastoma#study-download), (https://singlecell.broadinstitute.org/single_cell/study/SCP50/single-cell-rna-seq-analysis-of-astrocytoma), and (https://singlecell.broadinstitute.org/single_cell/study/SCP147/single-cell-analysis-in-pediatric-midline-gliomas-with-histone-h3k27m-mutation#study-visualize) (26) to explore the distribution of *MK3* from the tumor samples.

### Immune Cell Infiltration Analysis

The immune cell infiltration levels from tumors were quantified by the single-sample GSEA (ssGSEA) with the R package “GSVA” (27). The correlation between the *MK3* expression and immunoinhibitors, chemokines, and chemokine receptors across human cancers was determined on the Tumor-Immune System Interaction Database (TISIDB) website (http://cis.hku.hk/TISIDB/index.php). The correlation analysis between the expression of *MK3* and immunoinhibitors, chemokines, and chemokine receptor genes of the glioma was evaluated by using the R-package “ggplot2.”

### Tumor Immune Estimation Resource Database Analysis

The correlation of *MK3* expression with immune checkpoint expression from glioma was analyzed on the Tumor IMmune Estimation Resource (TIMER) website (https://cistrome.shinyapps.io/timer/) and adjusted by the tumor purity.

### Statistical Analysis

All gene expression data were normalized with log2 transformation. Statistical analyses were conducted by R (3.6.3). The survival data from the CGGA database were acquired by KM. The correlation analysis was evaluated by chi-square (χ^2^) test, Pearson’s correlation, or Spearman’s correlation analysis. Student’s t-test was used to determine the statistical significance of differences between groups, and the differences between more than two groups were analyzed by one-way ANOVA or the Kruskal–Wallis test. A two-sided Mann–Whitney test was used for non-parametric data. *p*-Value < 0.05 was considered statistically significant. *p < 0.05, **p < 0.01, and ***p < 0.001.

## Results

### The *MK3* Expression Analysis in Glioma

**Figure 1:**
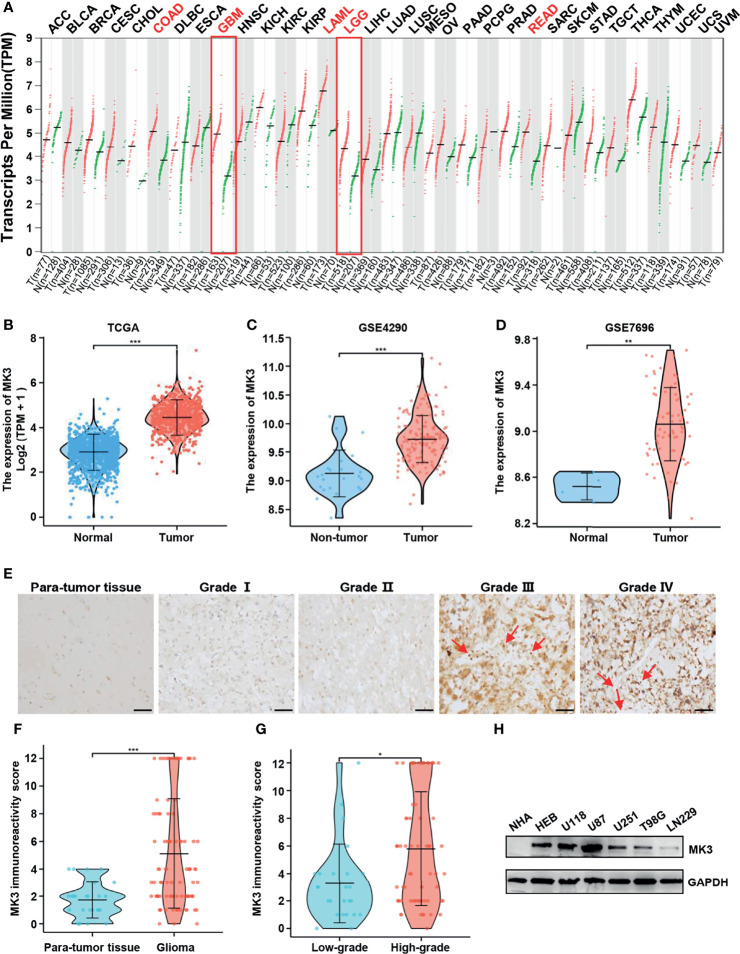
*MK3* was aberrantly upregulated in glioma. **(A)** *MK3* mRNA expression in different normal human tissues and tumor tissues from TCGA and the GTEx projects. **(B)** Comparison of *MK3* mRNA expression in GBM, LGG tissues (*n* = 1,157), and normal brain tissues (*n* = 689) from TCGA and GTEx databases. **(C)** Higher *MK3* mRNA expression was observed in glioma samples compared with non-tumor samples in GSE4290 dataset. **(D)** Higher *MK3* mRNA expression was observed in glioblastoma tissues compared with normal brain tissues in GSE7696 dataset. **(E)** The immunohistochemical staining of *MK3* in human glioma specimens. Scale bar: 50 μm. Red arrows indicate the immune cells. **(F)** The *MK3* immunoreactivity score of para-tumor tissues and glioma tissues. **(G)** The *MK3* immunoreactivity score of low-grade and high-grade glioma tissues. **(H)** The protein expression levels of *MK3* were examined in five glioma cell lines, HEB, and NHA human astrocytes by Western blotting analysis. GAPDH was used as loading control. TCGA, The Cancer Genome Atlas; GTEx, Genotype-Tissue Expression; GBM, glioblastoma; LGG, low-grade glioma; HEB, human normal brain glial cells; NHA, normal human astrocyte. *p < 0.05, **p < 0.01, and ***p < 0.001.

To determine the *MK3* expression in glioma, we analyzed the GEPIA database that contains the gene expression profile information across all tumor samples and paired normal tissues. The *MK3* was aberrantly overexpressed in GBM (*n* = 163) and LGG (*n* = 518) tissues compared with the normal brain tissues (*n* = 207) that had a low level of *MK3* mRNA expression among different normal human organs (**Figures 1A, B**). We further validated the data by using the GEO database; a similar overexpression pattern was observed in the glioma tissues compared with the non-tumor (GSE4290) or normal (GSE7696) tissues (**Figures 1C, D**). We also assessed the *MK3* expression levels in glioma tissues by immunohistochemistry (IHC) staining. The staining results showed a similar *MK3* expression pattern from the tumor tissues when compared with the para-tumor tissues (**Figures 1E, F**).

Furthermore, we measured the protein expression levels of *MK3* in five glioma cell lines (U118, U87, U251, T98G, and LN229), HEB, and NHA cell lines by Western blotting analysis. We observed that the protein levels of *MK3* were also higher in the majority of the glioma cell lines (**Figure 1H**). Collectively, our results suggest that *MK3* is aberrantly overexpressed in glioma.

### Correlation of *MK3* Expression With Clinicopathologic Features in Glioma

**Figure 2:**
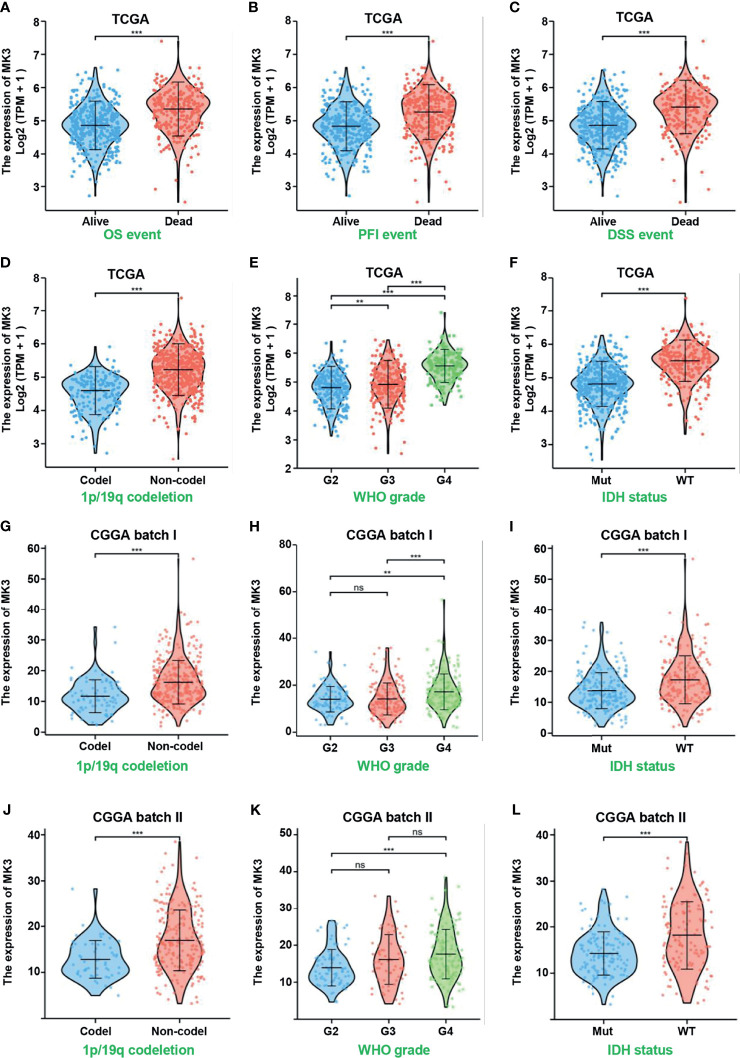
Overexpressed *MK3* was associated with poor clinicopathologic features of glioma. **(A–C)** *MK3* expression in OS **(A)**, PFI **(B)**, and DSS **(C)** events of human glioma samples in TCGA database. **(D–F)** Comparison of *MK3* expression in different groups of 1p/19q codeletion **(D)**, WHO grade **(E)**, and IDH status **(F)** of human glioma samples in TCGA database. **(G–L)** Comparison of *MK3* expression in different groups of 1p/19q codeletion **(G, J)**, WHO grade **(H, K)**, and IDH status **(I, L)** of human glioma samples in CGGA database. OS, overall survival; PFI, progression-free interval; DSS, disease-specific survival; TCGA, The Cancer Genome Atlas; CGGA, Chinese Glioma Genome Atlas. ns, no significance, **p < 0.01 and ***p < 0.001.

The significant positive correlation between the *MK3* level and WHO grade in glioma tissues (**Figures 1E, G**, **Table 1**) suggested a potential role of MK3 in the clinicopathologic features of glioma patients. To determine the clinical implication of *MK3* expression, we segmented the patients of GBM and LGG in TCGA database into low or high *MK3* expression groups according to the median value. As shown in **Table 2**, *MK3* overexpression was significantly correlated with the WHO grade, IDH status, 1p/19q codeletion, and age. We also analyzed the expression of *MK3* with OS, progression-free interval (PFI), and disease-specific survival (DSS) event of GBM and LGG, and the results showed that the expression of *MK3* was lower from alive patients (**Figures 2A–C**). Moreover, we determined the clinical implication of *MK3* expression from human GBM and LGG samples in TCGA database, and the significant differences were found in 1p/19q codeletion (**Figure 2D**), WHO grade (**Figure 2E**), and IDH status (**Figure 2F**). The results were also validated with the CGGA database (**Figures 2G–L**). Altogether, we demonstrate that *MK3* expression is closely correlated with the poor clinicopathologic features of glioma patients.

**Table 1:** *MK3* IHC staining and clinicopathologic characteristics of glioma patients.

Variable	Number (N)	MK3 staining			
		Low (%)	High (%)	*X* ^2^	*p*
**Sex**				1.094	0.296
Male	59	37 (62.7)	22 (37.3)		
Female	33	17 (51.5)	16 (48.5)		
**Age**				0.042	0.838
<50 years	40	23 (57.5)	17 (42.5)		
≥50 years	52	31 (59.6)	21 (40.4)		
**Tumor size**				1.137	0.286
<5 cm	29	19 (65.5)	10 (34.5)		
≥5 cm	29	15 (51.7)	14 (48.3)		
**WHO grade**				9.067	0.003
Low (I–II)	25	21 (84.0)	4 (16.0)		
High (III–IV)	67	33 (49.3)	34 (50.7)		

IHC, immunohistochemistry.

**Table 2:** The association between *MK3* expression and the clinical parameters in glioma patients in TCGA.

Characteristic	Low expression of MK3	High expression of MK3	*p*
***n***	348	348	
**WHO grade, *n* (%)**			<0.001
G2	151 (23.8%)	73 (11.5%)	
G3	135 (21.3%)	108 (17%)	
G4	26 (4.1%)	142 (22.4%)	
**IDH status, *n* (%)**			<0.001
WT	47 (6.9%)	199 (29%)	
Mut	296 (43.1%)	144 (21%)	
**1p/19q codeletion, *n* (%)**			<0.001
Codel	147 (21.3%)	24 (3.5%)	
Non-codel	199 (28.9%)	319 (46.3%)	
**Age, median (IQR)**	41 (33, 54)	50.5 (36, 62)	<0.001

TCGA, The Cancer Genome Atlas; IQR, interquartile range.

### The Prognostic Value of *MK3* in Glioma

**Figure 3:**
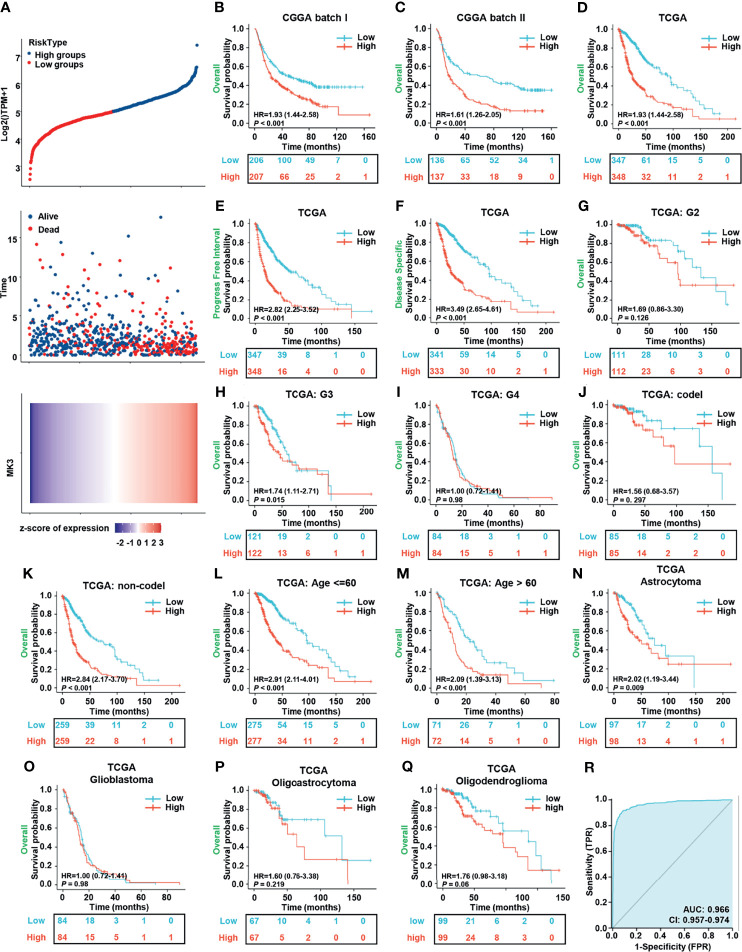
Overexpression of *MK3* was closely related to the poor prognosis of glioma patients. **(A)** The relationship between survival time, survival status of glioma patients, and *MK3* expression in TCGA dataset. Top: the curve of risk score. The dotted line represents the median risk score and divided the patients into low-risk and high-risk groups. Median: scatter plot distribution of survival time and survival status corresponding to the *MK3* expression of different samples. Bottom: heatmap of *MK3* expression. **(B, C)** The KM survival analysis for the correlation of *MK3* with overall survival of glioma patients in CGGA batch I **(B)** and batch II **(C)** datasets. **(D–F)** The KM survival analysis for the correlation of *MK3* expression with OS **(D)**, PFI **(E)**, and DSS **(F)** of glioma patients in TCGA dataset. **(G–Q)** Different subgroup analyses of KM for overall survival including WHO grade G2 **(G)**, G3 **(H)**, G4 **(I)**, and 1p/19q codeletion status: codel **(J)**, non-codel **(K)**, age less than or equal to 60 years **(L)**, age greater than 60 years **(M)**, and histological type astrocytoma **(N)**, glioblastoma **(O)**, oligoastrocytoma **(P)**, and oligodendroglioma **(Q)** of glioma patients in TCGA dataset. **(R)** ROC curve of GBM, LGG tissues (*n* = 1,157), and normal brain tissues (*n* = 689) from TCGA and GTEx databases for validating the diagnostic value of *MK3* in glioma patients. TCGA, The Cancer Genome Atlas; KM, Kaplan–Meier; CGGA, Chinese Glioma Genome Atlas; OS, overall survival; PFI, progression-free interval; DSS, disease-specific survival; ROC, receiver operating characteristic; GBM, glioblastoma; LGG, low-grade glioma; GTEx, Genotype-Tissue Expression.

To investigate the relationship between the *MK3* expression and the prognosis of glioma patients, we divided the cancer cases into high-risk and low-risk groups according to the cutoff value of the median risk score. The poorer prognosis and higher death rate were observed in the high-risk group (**Figure 3A**). Glioma patients were also separated into the *MK3*-high and *MK3*-low groups based on the expression levels with the median value to generate a KM survival curve. The KM survival curve demonstrated that the OS rate of glioma patients in the high-expression group was significantly poorer than that in the low-expression group in both the CGGA and TCGA datasets (**Figures 3B–D**). The PFI and DSS rate of glioma patients were also significantly and negatively correlated with *MK3* expression in TCGA dataset (**Figures 3E, F**). In addition, we performed a subgroup survival analysis of OS with TCGA dataset, and we found that high expression of MK3 was associated with poor prognosis in WHO grade G3, non-codel 1p/19q codeletion, age less than or equal to 60 years, age greater than 60 years, and histological type astrocytoma subgroup of glioma (**Figures 3G–O**). We also conducted the ROC curve analysis to evaluate the diagnostic value of *MK3*, and the area under the curve (AUC) was 0.966, which indicated a high diagnostic value of *MK3* in glioma (**Figure 3R**).

To further evaluate the prognostic value of *MK3*, we performed the univariate and multivariate Cox regression analyses, which are the widely used approaches for identifying predictive biomarkers. Univariate Cox analysis unearthed that *MK3* was a high-risk factor (hazard ratio (95% CI) = 3.037 (2.349–3.297), *p* < 0.001). Moreover, WHO grade, IDH status, 1p/19q codeletion, primary therapy outcome, age, and *MK3* expression were all significantly correlated with poor OS (**Table 3**), which indicated their association with the OS in glioma. On the other hand, the multivariate Cox analysis demonstrated that only WHO grade, IDH status, primary therapy outcome, gender, and age were independent prognostic factors for glioma (**Table 3**). In conclusion, these results demonstrate that *MK3* might serve as a valuable prognostic biomarker for glioma patients.

**Table 3:** Univariate and multivariate Cox proportional hazards analyses of *MK3* expression and overall survival for glioma patients.

Characteristics	Total (N)	Univariate analysis		Multivariate analysis	
		Hazard ratio (95% CI)	*p*-Value	Hazard ratio (95% CI)	*p*-Value
**WHO grade**	634				
G2	223	Reference			
G3	243	2.999 (2.007–4.480)	<0.001	1.979 (1.257–3.117)	0.003
G4	168	18.615 (12.460–27.812)	<0.001	6.842 (2.174–21.535)	0.001
**IDH status**	685				
WT	246	Reference			
Mut	439	0.117 (0.090–0.152)	<0.001	0.495 (0.289–0.849)	0.011
**1p/19q codeletion**	688				
Codel	170	Reference			
Non-codel	518	4.428 (2.885–6.799)	<0.001	1.591 (0.911–2.779)	0.103
**Primary therapy outcome**	461				
CR	138	Reference			
PR	64	1.275 (0.442–3.680)	0.653	1.182 (0.352–3.974)	0.787
PD	112	7.500 (3.598–15.634)	<0.001	5.803 (2.696–12.492)	<0.001
SD	147	3.299 (1.528–7.123)	0.002	2.115 (0.939–4.765)	0.071
**Gender**	695				
Female	297	Reference			
Male	398	1.262 (0.988–1.610)	0.062	1.810 (1.156–2.832)	0.009
**Age**	695				
≤60	552	Reference			
>60	143	4.668 (3.598–6.056)	<0.001	4.216 (2.562–6.938)	<0.001
**MK3**	695				
Low	348	Reference			
High	347	3.037 (2.349–3.927)	<0.001	0.926 (0.587–1.460)	0.740

CR, complete response; PR, partial response; PD, progressive disease; SD, stable disease.

### Function and Pathway Enrichment Analyses of *MK3* in Glioma

**Figure 4:**
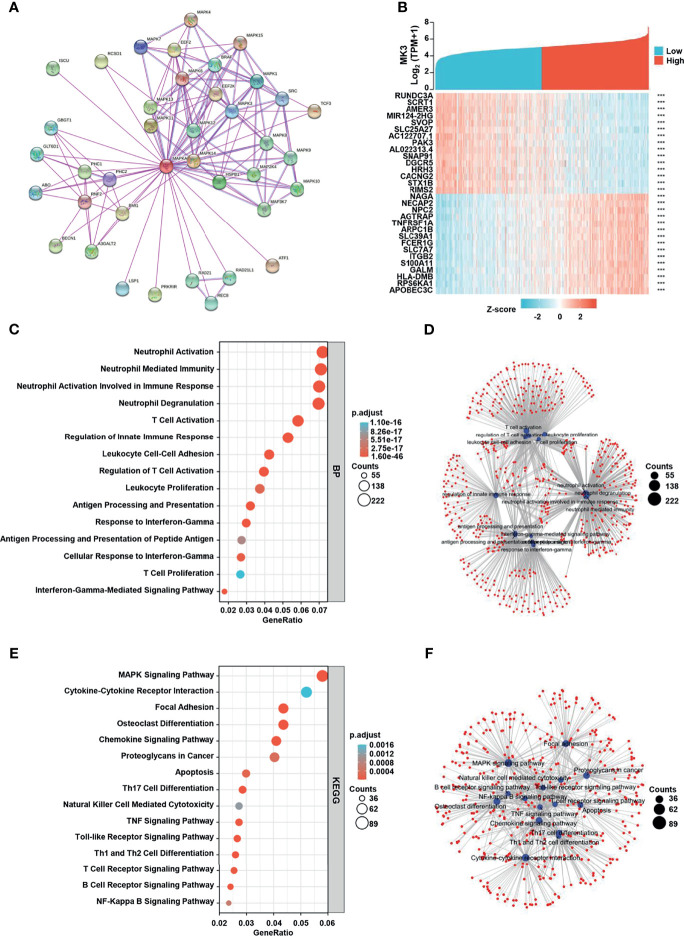
*MK3* related gene enrichment analysis. **(A)** The protein–protein interaction network analysis of *MK3* by using the STRING tool. **(B)** The top 15 genes with positive and negative correlations of *MK3* expression. **(C)** Based on the *MK3* related genes, GO analysis was performed. **(D)** The cnetplot of biological process in GO analysis. **(E)** KEGG pathway analysis of *MK3* related genes. **(F)** The cnetplot of KEGG pathway analysis. Data used for GO and KEGG analyses were obtained from TCGA. GO, Gene Ontology; KEGG, Kyoto Encyclopedia of Genes and Genome; TCGA, The Cancer Genome Atlas.

To identify the functional and physical interaction partners of *MK3*, we used STRING to analyze the protein–protein interactions. As shown in the interaction network (**Figure 4A**), a total of 38 *MK3* interaction proteins were found by experimental evidence. Furthermore, we recovered 3,067 positively correlated genes (Pearson’s correlation coefficient *r* > 0.4) and 1,229 negatively correlated genes (Pearson’s correlation coefficient *r* < −0.4) of *MK3* from TCGA transcriptome dataset. The top 15 positively correlated genes and negatively correlated genes of *MK3* were plotted in the heatmap (**Figure 4B**). To further uncover the potential role of *MK3* in glioma, we performed GO and KEGG analyses by TCGA dataset on *MK3* correlated genes. Interestingly, the results of GO classification demonstrated that *MK3* correlated genes were significantly enriched in immune-related functions among the top-ranked BPs, including the neutrophil activation, neutrophil-mediated immunity, neutrophil activation involved immune response, T-cell activation, regulation of innate immune response, leukocyte cell–cell adhesion, regulation of T-cell activation, leukocyte proliferation, antigen processing and presentation, response to interferon gamma, antigen processing and presentation of peptide antigen, cellular response to interferon gamma, T-cell proliferation, and interferon gamma-mediated signaling pathway (**Figures 4C, D**). KEGG pathway analysis showed that *MK3* correlated genes were also closely associated with immune-related signaling pathways, including cytokine–cytokine receptor interaction, osteoclast differentiation, chemokine signaling pathway, proteoglycans in cancer, Th17 cell differentiation, natural killer cell-mediated cytotoxicity, TNF signaling pathway, Toll-like receptor signaling pathway, Th1 and Th2 cell differentiation, T-cell receptor signaling pathway, B-cell receptor signaling pathway, and NF-kappa B signaling pathway (**Figures 4E, F**).

**Figure 5:**
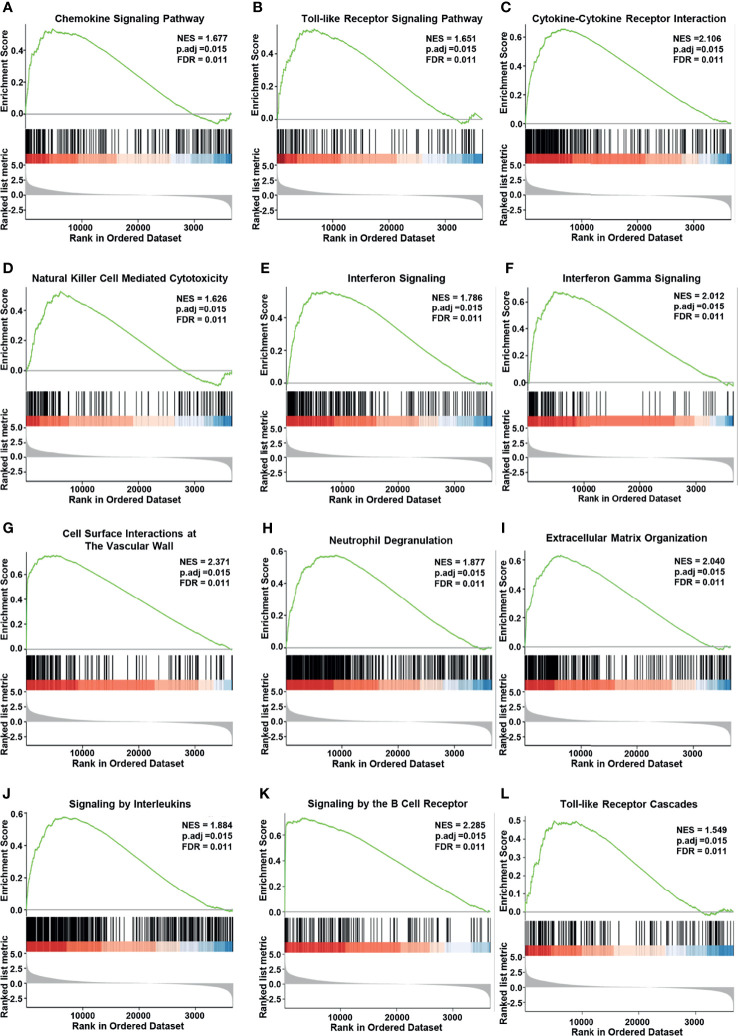
Enrichment plots from Gene Set Enrichment Analysis (GSEA). *MK3* upregulation was significantly correlated with “chemokine signaling” **(A)**, “Toll-like receptor signaling” **(B)**, “cytokine–cytokine receptor interaction” **(C)**, “natural killer cell-mediated cytotoxicity” **(D)**, “interferon signaling” **(E)**, “interferon-gamma signaling” **(F)**, “cell surface interactions at the vascular wall” **(G)**, “neutrophil degranulation” **(H)**, “extracellular matrix organization” **(I)**, “signaling by interleukins” **(J)**, “signaling by the B-cell receptor” **(K)**, and “Toll-like receptor cascades” pathways **(L)**. Data used for GSEA was obtained from TCGA. GO, Gene Ontology; KEGG, Kyoto Encyclopedia of Genes and Genome; TCGA, The Cancer Genome Atlas; GSEA, Gene Set Enrichment Analysis.

We also performed GSEA by using the *MK3*-low and *MK3*-high datasets from TCGA transcriptome dataset to identify signaling pathways that are affected by *MK3* overexpression in glioma. Gene sets related to chemokine signaling, Toll-like receptor signaling, cytokine–cytokine receptor interaction, natural killer cell-mediated cytotoxicity, interferon signaling, interferon gamma-signaling, cell surface interactions at the vascular wall, neutrophil degranulation, extracellular matrix organization, signaling by interleukins, signaling by the B-cell receptor, and Toll-like receptor cascades pathways were significantly enriched in the high *MK3* expression group (**Figures 5A–L**). Together, our results suggest that *MK3* might participate in the tumor immune microenvironment and immune regulation.

### Correlation Between *MK3* Expression and Immune Infiltration

**Figure 6:**
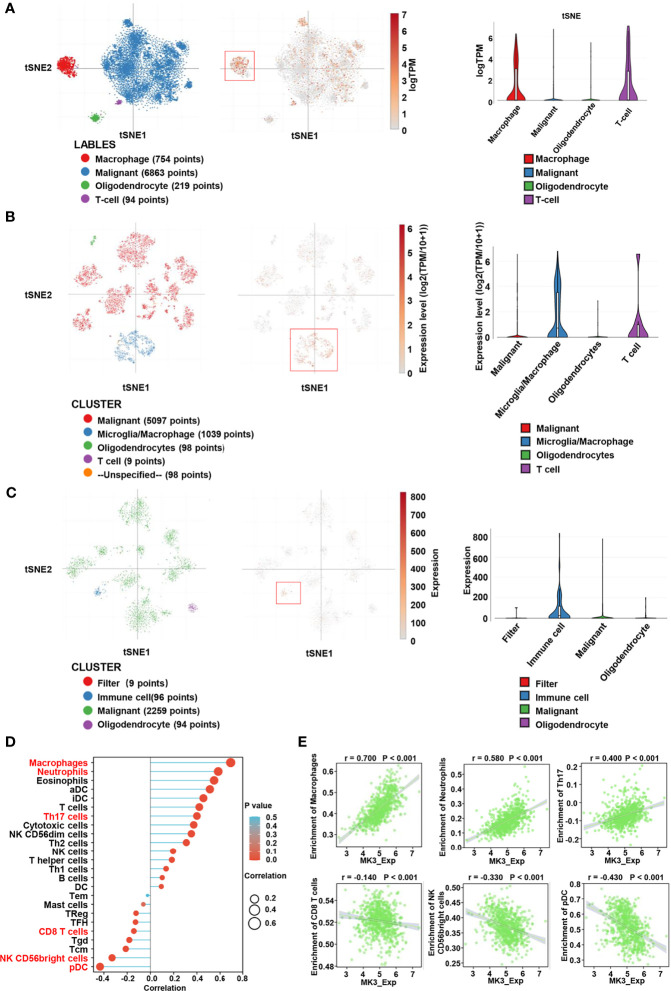
Correlation between *MK3* and immune cell infiltration. **(A)** Classification of single cells and the distribution of *MK3* in glioblastomas. Left: t-distribution stochastic neighbor embedding (tSNE) plot of all single cells. Cells are labeled based on the high expression of sets of marker genes for macrophage (red), malignant (blue), oligodendrocyte (green), and T cells (purple). Medium: tSNE plot of *MK3* specific expression cells (red). Right: violin plot of the distribution of *MK3*. **(B)** Classification of single cells and the distribution of *MK3* in tumor samples of IDH-mutant astrocytoma. Left: tSNE plot of all single cells. Cells are labeled based on the basis of high expression of sets of marker genes for malignant (red), microglia/macrophage (blue), oligodendrocyte (green), T cells (purple), and unspecified (yellow). Medium: tSNE plot of *MK3* specific expression cells (red). Right: violin plot of the distribution of *MK3*. **(C)** Classification of single cells and the distribution of *MK3* in gliomas with histone H3 lysine27-to-methionine mutations. Left: t-distribution stochastic neighbor embedding (tSNE) plot of all single cells. Cells are labeled based on the high expression of sets of marker genes for filter (red), immune cell (blue), malignant (green), and oligodendrocyte (purple). Medium: tSNE plot of *MK3* specific expression cells (red). Right: violin plot of the distribution of *MK3*. **(D)** Lollipop plots showing the correlation between the *MK3* expression level and the relative abundances of 24 immune cells in GBM and LGG samples from TCGA dataset. The size of dots shows the absolute value of Spearman’s R. **(E)** The correlation analysis between the expression of *MK3* and enrichment of immune cells (macrophages, neutrophils, Th17 cells, CD8 T cells, NK CD56bright cells, and plasmacytoid DC) in GBM and LGG samples from TCGA dataset. GBM, glioblastoma; LGG, low-grade glioma; TCGA, The Cancer Genome Atlas; DC, dendritic cells.

Considering the tumor heterogeneity, we further analyzed the distribution of *MK3* expression in glioma samples by assessing single-cell RNA-seq data. We observed that the extensive expression of *MK3* was in immune cells (macrophage and T cell) (**Figures 6A–C**), which further support our IHC staining data in glioma tissues (**Figure 1E**). These results suggest the potential role of *MK3* in regulating immune response. Therefore, we determined the correlation between *MK3* expression and immune infiltration in glioma by ssGSEA with Spearman’s R. We found that *MK3* expression was negatively correlated with infiltrating levels of T follicular helper (TFH), CD8 T cells, T gamma delta (Tgd), T central memory (Tcm), NK CD56bright cells, and plasmacytoid DC (pDC) cells and was positively correlated with infiltrating levels of macrophages, neutrophils, eosinophils, activated dendritic cells (aDC), immature DC (iDC), T, Th17, cytotoxic cells, NK CD56dim, Th2, NK, T helper, Th1, B cells, and DC (all *p* < 0.05, **Figures 6D, E**). These data indicate that *MK3* might participate in immune infiltration in glioma.

**Figure 7:**
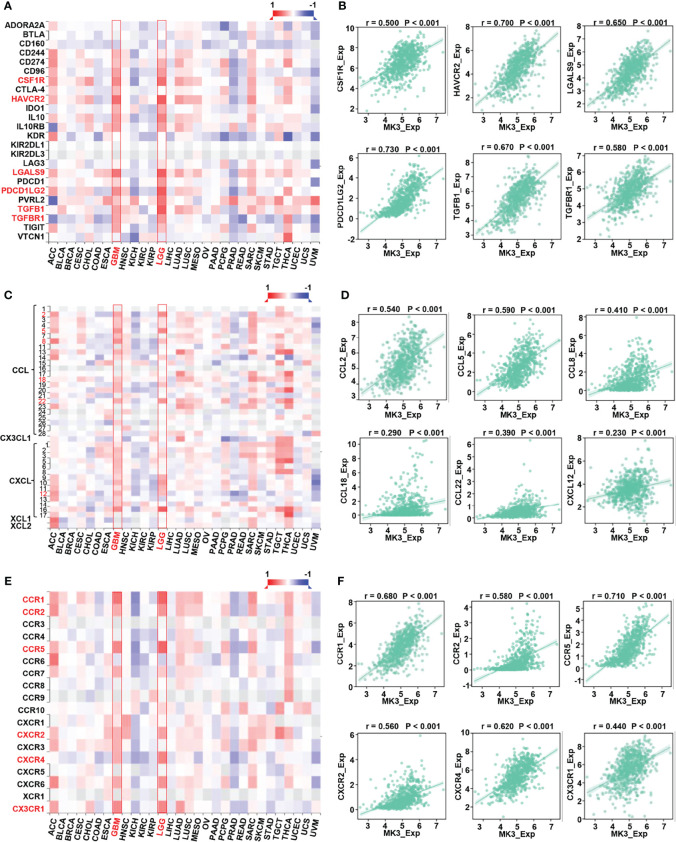
Correlation between *MK3* and immune regulated genes in glioma. **(A)** Correlation between the expression of *MK3* and immunoinhibitors across human cancers. **(B)** The correlation analysis between the expression of *MK3* and immunoinhibitor genes (*colony-stimulating factor 1 receptor*, *CSF1R*; *hepatitis A virus cellular receptor 2*, *HAVCR2*; *galectin 9*, *LGALS9*; *programmed cell death 1 ligand 2*, *PDCD1LG2*; *transforming growth factor beta 1*, *TGFB1*; and *transforming growth factor beta receptor 1*, *TGFBR1*) in GBM and LGG samples from TCGA dataset. **(C)** Correlation between the expression of *MK3* and chemokines across human cancers. **(D)** The correlation analysis between the expression of *MK3* and chemokine genes (*C-C motif chemokine ligand 2*, *CCL2*; *C-C motif chemokine ligand 5*, *CCL5*; *C-C motif chemokine ligand 8*, *CCL8*; *C-C motif chemokine ligand 18*, *CCL18*; *C-C motif chemokine ligand 22*, *CCL22*; and *C-X-C motif chemokine ligand 12*, *CXCL12*) in GBM and LGG samples from TCGA dataset. **(E)** Correlation between the expression of *MK3* and chemokine receptors across human cancers. **(F)** The correlation analysis between the expression of *MK3* and chemokine receptor genes (*C-C motif chemokine receptor 1*, *CCR1*; *C-C motif chemokine receptor 2*, *CCR2*; *C-C motif chemokine receptor 5*, *CCR5*; *C-X-C motif chemokine receptor 2*, *CXCR2*; *C-X-C motif chemokine receptor 4*, *CXCR4*; and *C-X3-C motif chemokine receptor 1*, *CX3CR1*) in GBM and LGG samples from TCGA dataset. GBM, glioblastoma; LGG, low-grade glioma; TCGA, The Cancer Genome Atlas.

Taking advantage of TISIDB website, we also explored the correlation between the expression of *MK3* and immune-related genes in glioma. Our results suggest that the expression of *MK3* was positively correlated to the majority of immunoinhibitors (**Figures 7A, B**), pro-tumor chemokines (**Figures 7C, D**), and chemokine receptors (**Figures 7E, F**) in both GBM and LGG patients.

**Figure 8:**
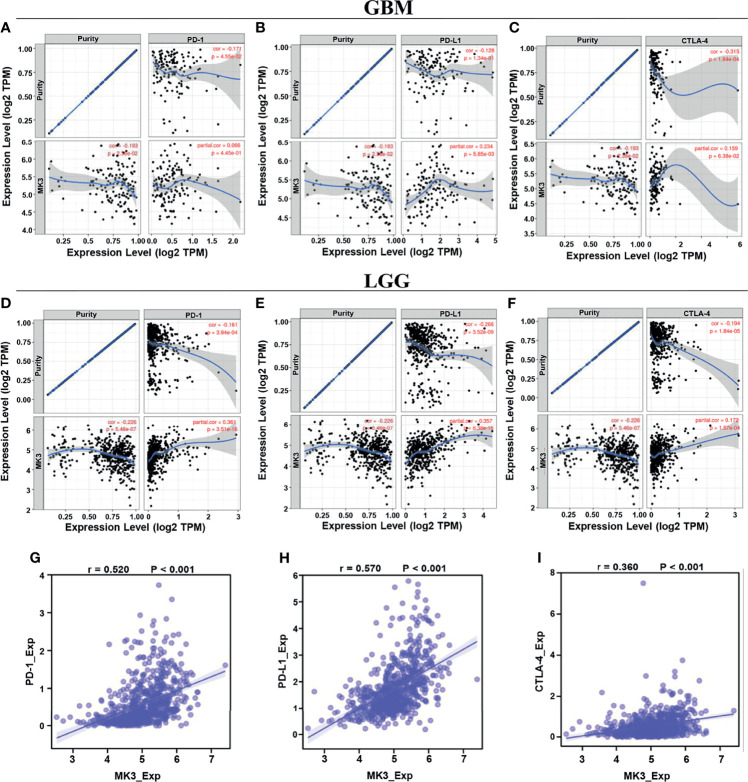
Correlation of *MK3* expression with *PD-1*, *PD-L1*, and *CTLA-4* in glioma. **(A–C)** Correlation of *MK3* expression with *PD-1* **(A)**, *PD-L1* **(B)**, and *CTLA-4* **(C)** in GBM adjusted by tumor purity using TIMER. **(D–F)** Correlation of *MK3* expression with *PD-1* **(D)**, *PD-L1* **(E)**, and *CTLA-4* **(F)** in LGG adjusted by tumor purity using TIMER. **(G–I)** The expression correlation of *MK3* with *PD-1* **(G)**, *PD-L1* **(H)**, and *CTLA-4* **(I)** in GBM and LGG samples from TCGA dataset. GBM, glioblastoma; TIMER, Tumor IMmune Estimation Resource; LGG, low-grade glioma; TCGA, The Cancer Genome Atlas.

*Programmed cell death protein 1* (*PD-1*/PDCD1), *programmed cell death 1 ligand 1* (*PD-L1*/CD274), and *cytotoxic T lymphocyte antigen 4* (*CTLA-4*) are vital immune checkpoints that play important roles in tumor immune escape. And they also served as predictive markers for the therapeutic efficacy of immune checkpoint inhibitors (ICIs). Thus, we especially assessed the relationship of *MK3* with *PD-1*, *PD-L1*, and *CTLA-4* in glioma by using TIMER tool. The results from TIMER website illustrated that there was a significant positive correlation between *MK3* expression and *PD-1*, *PD-L1*, and *CTLA-4* in glioma, which was adjusted by tumor purity (**Figures 8A–F**). Similarly, we also found that the expression of *MK3* was significantly positively correlated with *PD-1* (*r* = 0.520, *p* < 0.001), *PD-L1* (*r* = 0.570, *p* < 0.001), and *CTLA-4* (*r* = 0.360, *p* < 0.001) in glioma (**Figures 8G–I**). Taken together, these results suggest that *MK3* might participate in tumorigenesis and the development of glioma by regulating the tumor immune escape.

## Discussion

*MK2* and *MK3* are MAPK-activated proteins that are often being classified as isoenzymes. They have a high degree of structural similarity, and they also share activators, substrates, and physiological functions (28). Both are ubiquitously expressed in various tissues of mammals. However, *MK3* is mainly expressed in muscle, liver, heart, and T and NK cells (11). *MK2* and *MK3* usually cooperate to regulate a variety of cellular processes, such as cytokine production, endocytosis, cytoskeleton organization, cell migration, cell cycle control, chromatin remodeling, and gene expression (28), while the potential functions and effects of *MK3* for tumorigenesis are poorly understood. One study suggested that *MK3* could act as a reliable prognostic indicator in colorectal cancer patients (25), but the expression, clinical significance, and underlying molecular mechanisms of *MK3* in glioma have yet not been reported.

In this study, we found that the *MK3* was lower in the normal brain tissues and differentially expressed in many types of cancers. Importantly, *MK3* expression was markedly elevated in GBM and LGG, which was further validated by using the GEO and CGGA database and the Western blotting approach in glioma cell lines. In addition, IHC staining results showed a similar result in glioma tissues (**Figure 1**). We also demonstrated that the overexpression of *MK3* was closely associated with WHO grade, 1p/19q codeletion, IDH status, and age. We further validate that the higher *MK3* expression correlated with the poor clinicopathologic features of glioma (**Figure 2**). Moreover, the KM survival curve analyses showed that a high level of *MK3* was correlated with poor prognosis in glioma from both TCGA and CGGA datasets (**Figure 3**). Together, our findings reveal the novel roles of *MK3* for glioma and define *MK3* as a valuable biomarker.

The survival analyses found that high expression of *MK3* was negatively correlated with the OS rate of glioma patients (**Figures 3B–D**), while survival analyses of histological type for glioma patients showed that *MK3* did not affect survival in GBM (**Figure 3O**), which might be limited by the small number of patients enrolled due to the short survival time and low survival rate of GBM (29). More GBM cases were needed to further evaluate the prognostic value of *MK3* in GBM in the future.

Multivariate Cox analysis demonstrated that gender was an independent prognostic factor for glioma (**Table 3**). Sex differences have been well identified in many brain tumors including glioma. The sex-specific effects for the incidence, phenotype, and outcome of glioma have been well described; however, few insights are available to distinguish male and female glioma patients at the molecular level or allow specific targeting of these biological differences (30, 31). More studies are needed to focus on sex differences in GBM in terms of pathophysiology, hormones, metabolism, tumor location, treatment response, recurrence, and outcome (31).

Our function and pathway enrichment analyses suggested that *MK3* co-related genes enriched in interferon-gamma signaling pathway (**Figures 4**, **5**), which is consistent with previous studies that *MK3* regulated the transcription activity of type I interferon-dependent genes (20) and that *MK3* could suppress interferon-gamma expression to control NK cell cytotoxicity and Th1 CD4 T-cell development (23). Interferon has been widely used in the clinic for cancer treatment, as the interferon signaling pathway regulates the immune checkpoint blockade and tumor microenvironment (32). This study also found that correlated genes of *MK3* in glioma were mainly associated with tumor immune regulations. Moreover, single-cell RNA-seq data revealed that *MK3* was expressed in immune cells (**Figures 6A–C**). All these results indicated the potential roles of *MK3* in regulating tumor immunity in glioma.

Although *MK3* has been reported to express in NK and T cells, as well as regulate the inflammatory response of macrophages (33) and neutrophil recruitment (13), the exact functions and possible mechanisms of *MK3* for regulating tumor immunity are unclarified. We found that *MK3* expression was significantly correlated with infiltration of various immune cells, such as macrophages, neutrophils, Th17, CD8 T cells, NK CD56bright cells, and pDC (**Figures 6C, D** **)**. Immune infiltrations have been shown associated with the prognosis of a patient (34), which suggested that *MK3* may regulate immune infiltration and, in turn, affects glioma prognosis. More work is still needed to explore the special role of *MK3* in tumor immunity of glioma.

Immunoinhibitors, such as *PD-1*, *PD-L1*, and *CTLA-4*, have gained widespread attention, as they can serve as immune checkpoint targets in multiple cancers to block immunoinhibitory signals and enable to produce effective antitumor responses (35). Our study demonstrated that the expression of *MK3* was positively correlated with *PD-1*, *PD-L1*, and *CTLA-4* (**Figure 8**), suggesting a prominent role of *MK3* in regulating the expression of immune checkpoints and immunotherapy. Future investigations of *MK3* should advance the therapeutic efficiency for glioma.

Chemokines are the largest subfamily of cytokines that could recruit different immune cell subsets into the microenvironment *via* interactions with chemokine receptors, which regulate tumor progression and therapeutic outcomes (36). Here, we reported that *MK3* expression was positively correlated with the majority of chemokines and chemokine receptors, such as *CCL* (2, 5, 8, 18, 22), *CXCL12*, *CCR* (1, 2, 5), *CXCR2*, *CXCR4*, and *CX3CR1* (**Figures 7C–E**), which play pro-tumor roles. Thus, our findings highlight the essential roles of *MK3* in immune infiltrations in glioma.

In summary, we found that MK3 was significantly aberrantly upregulated in glioma. Through a series of comprehensive approaches, we demonstrated that the increased *MK3* expression is strongly associated with clinicopathologic features, poor prognosis, and immune cell infiltration in glioma. Altogether, our results suggest that *MK3* might serve as a valuable prognostic factor and a promising novel immunotherapy target for glioma.

## Data Availability Statement

The original contributions presented in the study are included in the article/supplementary material. Further inquiries can be directed to the corresponding authors.

## Ethics Statement

The studies involving human participants were reviewed and approved by the Affiliated Hospital of Xuzhou Medical University. The patients/participants provided their written informed consent to participate in this study.

## Author Contributions

HC and JR designed the study. JR, JS, ML, and ZZ performed the research. JR and HC analyzed the data. JR, DY, and HC wrote the paper.

## Funding

We are grateful for the support from grants from the Research Foundation of Xuzhou Medical University (53681921), National Natural Science Foundation of China (82002516), the Natural Science Foundation of Jiangsu Province in China (Grant No. BK20190984), and the Natural Science Fund for Colleges and Universities in Jiangsu Province (19KJB310023).

## Conflict of Interest

The authors declare that the research was conducted in the absence of any commercial or financial relationships that could be construed as a potential conflict of interest.

## Publisher’s Note

All claims expressed in this article are solely those of the authors and do not necessarily represent those of their affiliated organizations, or those of the publisher, the editors and the reviewers. Any product that may be evaluated in this article, or claim that may be made by its manufacturer, is not guaranteed or endorsed by the publisher.
